# Preferences, use of and satisfaction with mental health services among a sample of Puerto Rican cancer patients

**DOI:** 10.1371/journal.pone.0216127

**Published:** 2019-07-18

**Authors:** Eida M. Castro-Figueroa, Normarie Torres-Blasco, Milagros C. Rosal, Wallesca P. Castro, Marilis E. González-Lorenzo, Héctor Vélez, Rosario Costas-Muñiz, Julio C. Jiménez

**Affiliations:** 1 School of Behavioral and Brain Sciences, Ponce Research Institute, Ponce Health Sciences University, Ponce, Puerto Rico; 2 Department of Psychiatry, Ponce Research Institute, Ponce Health Sciences University, Ponce, Puerto Rico; 3 Division of Preventive and Behavioral Medicine, Department of Medicine, University of Massachusetts Medical School, Worcester, Massachusetts, United Sates of America; 4 School of Medicine, Ponce Research Institute, Ponce Health Sciences University, Ponce, Puerto Rico; 5 Ponce Hematology Oncology, Ponce, Puerto Rico; 6 Department of Psychiatry and Behavioral Sciences, Memorial Sloan Kettering Cancer Center, New York, New York, United Sates of America; University of Auckland, NEW ZEALAND

## Abstract

**Objective:**

The purpose of this study was to describe the preferences, the use, satisfaction of mental health services (MHS) among a sample of Puerto Rican patients with cancer undergoing oncology treatment.

**Methods:**

A convenience sample of 120 patients diagnosed with cancer was recruited. Self-report questionnaires assessed socio-demographic and background questions, and the Mental Health Service Preference, Utilization and Satisfaction Questionnaire (MHSPUS). The Socio-demographic and Background Questionnaire inquired about participants’ demographic and socioeconomic characteristics, and included questions such as history of psychiatric diagnosis and spiritual practices. Univariate and Chi square analyses were used for descriptive purposes. Logistic regressions were used to explore associations between sociodemographic factors and MHS preferences and use.

**Results:**

The majority of the sample were females (53.8%), 61 and older (53.8%), and married or living with partner (57.1%), and reported an income equal to or less than $12,000 per year (44.4%), which places them under the US federal poverty line. Most of the participants (66.7%) reported being receptive to seeking services. Findings showed a significant association between living situation and past (p < .05) and lifetime use (p < .05) of MHS and past use of MHS. Participants living alone were more like to have used MHS in the past and during their lifetime. Adjusted logistic regression analyses revealed that living with someone was a protective factor for not using MHS in their lifetime (OR = 0.28; C1 = 0.08–0.95). Participants preferred to receive MHS at the oncology clinic, preferably on the date of their oncology appointments and during morning hours.

**Conclusion:**

Findings support the integration of mental health services within the oncology practice setting.

## Introduction

In Puerto Rico, cancer incidence increased significantly between 2007 and 2011,[1] with cancer becoming the first cause of death among the adult population in the island.[2] A cancer diagnosis represents a physical and emotional burden to patients.[3]^,^[4]^,^[5] Within the cancer care continuum, patients experience emotional distress in the form of fear, anxiety and sadness[6] and 30–75% of patients with cancer experience some emotional distress.[7] These emotional reactions are often associated with the perception of cancer as a fatalistic event.[8]

In the United States, Hispanic patients may be at higher risk of experiencing high levels of depressive symptoms and emotional distress and poor quality of life when compared to other minority ethnic groups.[9]^,^[10] Furthermore, emotional distress experienced by patients with cancer may also evolve into a psychiatric disorder.[11] To this end, a 2011 systematic review[12] found a high prevalence of Major Depressive Disorder (16.3%, IC of 95%: 13.4–19.5); Adjustment Disorder was (19.4%, IC of 95%: 114.5 to 24.8) and Anxiety Disorders (10.3%, IC of 95%: 5.1 to 17.0) among patients with cancer. A study conducted in Puerto Rico targeting cancer patients hospitalized at a general hospital (n = 206) revealed that 65% of the sample (referred by physicians for psychological evaluation) were diagnosed with a psychiatry disorder.[13] Among those diagnosed, 43.9% met the clinical criteria for a Mood Disorders and 26% presented symptoms of an Adjustment Disorder.[13]

In Puerto Rico a series of community-based participatory research initiatives have identified unmet emotional support needs among cancer patients and survivors.[14]^,^[15] Furthermore, a needs assessment conducted with Puerto Rican cancer patients under oncology treatment (n = 103) found that 34% reported unmet psychosocial needs related to “feeling depressed”.[16] Also, a study[17] aimed at identifying cancer clinical and support services resources in southern Puerto Rico mapped clinical (e.g. oncology clinics and primary care), support services (e.g. American Cancer Society, support groups) and combined services (e.g. clinical + support). Regarding referral practices, clinical service organizations received most of their referral from medical clinical services (77%); 56% of the referrals received by support service organizations came from clinical organizations and all combined service organizations (100%) received referrals from clinical service organizations. Percentage of agencies reporting received referrals from support service organizations across the different groups were: 56% for clinical (including clinical mental health services), 68% for support and 58% for combined. These data confirm that there is a variety of clinical, support and combined service resources available in the geographic area with established referral practices. However, little is known about Puerto Rican cancer patient-level factors related to the preferences, use of and satisfaction with mental health services.

Considering that symptoms of depression, anxiety and emotional distress are common among patients diagnosed with cancer,[18] specialized mental health services should be considered an essential component of their treatment, and such services should be integrated into the cancer care continuum,[19] as recommended by a decade-old Institute of Medicine (IOM) report.[20] However, the extent of the implementation of these recommendations and the quality of psychosocial care offered to the cancer population are unknown.[21] For example, a survey of oncologists (n = 448) affiliated with the American Society for Clinical Oncology (ASCO) found that only 47% of them reported referring their patients to mental health services and only 50% of participating physicians had mental health services affiliated to their practice.[22] Similar findings were reported by Castro and collaborators[17] from a study conducted in the southern area of ​​Puerto Rico. This study evaluated collaborative practices among clinical and support organizations providing services to patients with cancer and cancer survivors (n = 56). The study revealed that 43% of the surveyed entities generated mental health service referrals for patients and cancer survivors.[17] Also, there were gaps in the availability of specialized psychosocial care for patients with cancer.[17] A study conducted by Costas-Muñiz and collaborators[23] reveal that only 31% of Latina breast cancer survivors reported having a discussion with their cancer care provider about their emotional care needs and were less likely to have contact with mental health professionals (OR D 0.42, CI D 0.19–0.94). There is a lack of culturally tailored psychosocial care services for patients with cancer.[24]

Based on the overarching need to provide high quality specialized mental health services to patients with cancer in Puerto Rico, we investigated the preferences, use of and satisfaction with health services in a sample of patients with cancer undergoing oncology treatment in Southern Puerto Rico. This study sought to: 1) Describe the preferences for, use of, and satisfaction with mental health services in a sample of Puerto Rican patients with cancer undergoing oncologic treatment; 2) Evaluate socio-demographic factors associated with mental health service preferences and use; and 3) Explore sociodemographic factors predicting mental health service use.

## Materials and methods

### Study design

The study employed a descriptive cross-sectional design with the purpose of obtaining current information about the preferences, use of and satisfaction with mental health services. A convenience sample of 120 Puerto Rican cancer patients, from the southern region of Puerto Rico, participated in the study. Given the sampling method chosen (convenience sampling) is not probabilistic, the results cannot be generalized. The sample of 120 participants represent 5.13% of sthe average annual cancer cases in the southern (Ponce) region of Puerto Rico (ten-year average cases = 2,337[25])

### Ethics statement

Ethical approval was granted by the Ponce Health Sciences University-Ponce Research Institute (protocols number 141007-EC and 140917-EC). Prior to data collection, the study was explained to the participant and an opportunity to ask questions was provided. Written informed consent was obtained from each participant. Confidentiality was kept by using a unique study code number to identify research records for each subject. All physical study data is kept in locked storage indefinitely and all electronic study data is password protected with access restricted to approved personnel.

### Recruitment

Recruitment process involved arranging verbal collaborative agreements among cancer support groups and community oncology clinics. Cancer support groups that collaborated with recruitment process are peer-led (usually a cancer survivor) and offer social and informational support. Oncology clinics where recruitment also took place were ambulatory private clinics located in an urban area that provided general oncology care to patients from inner-city and rural areas of southern Puerto Rico. Estimates of participants recruited in support groups versus oncology clinic were not documented. Support group leaders and administrative personnel of community oncology clinics provided informative flyers to potential participants. Individuals interested in participating were screened for eligibility by a research assistant.

### Participants

Individuals were eligible to participate in the study if they had a cancer diagnosis, were undergoing active oncology treatment (chemotherapy, radiotherapy), and were cognitively capable of providing voluntarily informed consent. Those meeting eligibility were consented in writing prior to completing the study assessment.

### Instruments

Participants completed a packet of self-report tools that included socio-demographic and background questions and the Mental Health Service Preference, Utilization and Satisfaction Questionnaire (MHSPUS) developed by Dr. Eida Castro (lead author). The MHSPUS questionnaire was developed by the study's lead author using existing literature[26–29] and observations from Dr. Castro’s past clinical experience with cancer patients. After questionnaire items were created, they were subject content validity process by a panel of experts in the field (n = 3, as recommended elsewhere[30]). The panel consisted of a psychiatrist experienced in the area of psychosomatic medicine and director of the PHSU Health Psychology Training and Research and the Health Psychology Clinical Services program in general hospitals, extending services to cancer patients. Another expert was a clinical psychologist and behavioral medicine scientist expert on the subject of mental health service utilization of Hispanic patients diagnosed with chronic diseases. The third expert was a clinical psychologist and expert in the area of ​​test construction. The panel of experts evaluated the adequacy and relevance of the MHSPUS item using an evaluation guide designed by Dr. Castro based on the Lawshe Content Validity Index.[30,31] The MHSPUS questionnaire consists of 3 sections and 22 items. The first section asks about the use of and satisfaction with mental health services in the present and in the past (yes or no). Also, it includes an item asking if participants have ever felt the need to receive mental health services but refrained from seeking those services. Participants with no previous history of mental health services utilization were asked about their receptivity to seek mental health services if needed in the future (yes or no) and, if yes, to choose which type of service they would prefer using. The second section inquiries about the respondent’s preferences for mental health services in terms of type of service (e.g. psychological, psychiatric, counseling, social work), modality (e.g. individual, group, family), days and times of services, frequency of appointments, and location(s). Participants chose all that applied from a list of services. The last section assesses access to and use smartphones with internet access, internet use, email use, as well as, their interest in receiving mental health information over the smart phone with internet access. Socio-demographic, clinical and background questions assessed sex, age, education, marital status, living situation (accompanied of alone), employment status, medical insurance, income and one question assessing perceived financial burden (e.g. is income enough to cover all expenses?). We only assessed type of cancer tumor as clinical characteristic. No tumor stage was assessed because, due to the nature of the study data collection process, we did not have access to all patients’ medical records in order to accurately assess this variable. Background questions included personal and family psychiatric history and spiritual practices using a Spanish modified version of the FICA Spiritual Assessment Tool.[32]^,^[33] Spiritual practices was assessed as a potential confounder given the tendency of cancer patients to incorporate such practices through the cancer care,[34–38] also because Hispanics may prefer religious practices to cope with emotional adversity.^34^ and a barrier to seeking psychiatric services^35^

### Procedure

The study was approved by the Ponce Research Institute Institutional Review Board (PRI-IRB). After signing informed consent, participants were provided with a pocket of self-report instruments to complete. The process took approximately 25 minutes. Although the instruments were designed as self-report measures, an undetermined number of subjects asked to be assisted with the process by the research personnel collecting the data. The reasons to be assisted given by participants were not having eye glasses, unable to read and write and, unable to write due to hand neuropathy. Participants were provided with a $25 incentive for their participation.

### Data analysis

Study data were analyzed in SPSS version 21 (SPSS Institute, Chicago, Illinois) for further analysis. Univariate analysis (frequencies, mean and standard deviation) were used to describe current and past use of mental health services, preferences for and satisfaction with such services, as well as access to and receptivity to receive mental Health information through electronic devices. Additionally, descriptive analyses were performed to report sociodemographic, clinical, and background characteristics. Chi square analysis were used to assess relationship among all measured socio-demographic factors and use of and preferences with mental health services, along with the use of electronic devices for mental health information and services. Then, we applied the same analysis to explore differences by sex, age (sixty and younger and older than sixty), marital status (with or without partner), income ($0-$19,000, $19,001 –$35,000, $35,000 and up), employment status (employed and not employed), education (less than HS, HS and technical degree, undergraduate degree and up), living situation (accompanied and alone) and medical insurance (government insurance, private insurance and combined government/private insurance). We used a two-sided significance level of a = 0.05.

Unadjusted and adjusted regression analyses were computed to further explore associations among sociodemographic factors and MHS use, as well as, predictors of current, past and lifetime use of MHS. The dependent variable in the model were current MHS use (yes/no), past MHS use (yes/no), and lifetime MHS use (yes/no). Lifetime MHS use was transformed by combining current and past MHS use. Also, categorical variables were transformed as dummy variables. The independent variables included as covariates were living situation, age, partnered, education, employment status, medical insurance, income and income enough to satisfy cost of living. Since less than 7% of the data was missing, and assuming that this data is missing completely at random (MCAR), we used listwise deletion method to handle missing data. A two-sided significance level of *p*< .05 was considered statistically significant.

## Results

Table 1 presents the socio demographic and clinical characteristics of the sample. The majority of the sample were females (54.2%, n = 65), 66 and older (n = 42, 35%), married (n = 50, 41.7%) and living with partner (n = 45, 37.5%). 30.8% (n = 37) reported high school as their highest education achievement, 57.8% were retired (n = 67) and 44.5% (n = 53) reported an income equal to or less than $12,000 per year, which places them under the US federal poverty line. The most common type of cancers among participants were: breast cancer: 30.8% (n = 37), lymphoma: 10.8% (n = 13), colon cancer: 10% (n = 12) and prostate: 8.3% (n = 10).

**Table 1:** Sociodemographic and clinical characteristics (n = 120).

Variable	Frequency	%
**Sex**		
Male	55	45.8
Female	65	54.2
**Age Range**		
21–25	5	4.2
26–30	1	0.8
31–35	0	0.0
36–40	2	1.7
41–45	7	5.8
46–50	9	7.5
51–55	13	10.8
56–60	16	13.3
61–65	21	17.5
66	42	35.0
Missing	4	3.3
**Marital Status**		
Single	19	15.8
Married	50	41.7
Living with partner	14	11.7
Divorced	17	14.2
Separated	2	1.7
Widow	16	13.6
Missing	2	1.7
**Living with**		
Spouse	45	37.5
Other family member	4	3.3
Parents	8	6.6
Alone	21	17.5
Siblings	4	3.3
Adults siblings	11	9.16
Spouse & adult’s siblings	17	14.16
Other	2	1.6
Spouse & siblings	2	1.6
Spouse, siblings & other	1	.8
Parents & siblings	3	2.5
Spouse, siblings & adults’ siblings	2	1.6
**Labor status**		
Employed full time	12	10
Employed part time	4	3.3
Temporary employment	4	3.3
Retired	67	55.8
Student	2	1.7
Unemployed	18	15.0
Disability status	7	5.8
Retired & Disable	1	.8
Other	2	1.7
Temporary job & unemployed	1	.8
Retired & Unemployed	1	.8
Student & Unemployed	1	.8
**Education**		
Did not attend	32	26.7
High school	37	30.8
Technical degree	23	19.2
College Degree	19	23.3
**Annual Income**		
Up to $12,000	53	44.5
$12,001–19,000	33	27.5
$19,001–35,000	23	19.2
$35,001–60,000	7	15.8
$60,001–100,000	2	1.7
$100,001–250,000	1	.8
Missing	1	.8
**Type of cancer**		
Bone marrow	2	1.6
Head/ Neck	5	4.1
Colon/ Liver	4	3.3
Prostatic	10	8.3
Cervical	2	1.6
Colon	12	10
Stomach	3	2.5
Liver	3	2.5
Lymphoma	13	10.8
Breast	37	30.8
Medulla	2	1.6
Pulmonary	5	4.1
Myeloma	3	2.5
Rectal	2	1.6
Kidney	3	2.5
Pancreas	4	3.3
Ovary	3	2.5
Other*	7	5.8

*Brest & bone, Soft tissue, Testicular, Intestine, Larynx, Leukemia & Pelvic.

### Mental health service preferences use and satisfaction

Table 2 summarizes mental health service utilization and satisfaction. Descriptive analysis reveals that 13.7% (n = 16) of participants were receiving mental health services. Twenty-five percent (n = 31) of the sample reported having used mental health services in the past. For those who have used mental health services, the majority reported using only psychological services (n = 10, 62.6%). Satisfaction with mental health service use was explored among those reporting receiving such services (See Table 2). Among participants who reported receiving mental health services at the time of the study (N = 16), the majority reported being very satisfied (n = 8, 50%), very unsatisfied (n = 7, 22.5%) and followed by nor satisfied or unsatisfied (n = 5, 16.4). Moreover, of those who reported having received mental health services in the past (N = 31), 29% (n = 9) of participants reported being very satisfied, 22.5% (n = 7) reported being very unsatisfied, and followed by being nor satisfied or unsatisfied, 16.4% (n = 5)and, 12.9% (n = 4) of those receiving this service in the past decided not to answer the question.

**Table 2:** Mental health service (MHS) use and satisfaction (n = 120).

Variable	Frequency	%
**Currently receiving MHS**		
Yes	16	13.7
No	104	86.7
**In the past received MHS**		
Yes	31	25.8
No	87	72.5
Missing	5	1.7
**Felt the need to receive MHS but reframed**		
Yes	13	11.1
**Variable**	**Frequency**	**%**
No	99	84.6
Missing	5	4.2
**Type of MHS receiving now**		
Psychological	10	62.6
Psychiatric	4	25
Psychiatric & social work	1	6.2
Missing	1	6.2
**Type of Therapy**		
Individual Therapy	14	87.6
Family Therapy	1	6.2
Missing	1	6.2
**Satisfaction with MHS receiving now**		
Very satisfied	8	50
Moderate satisfied	3	18.8
Neutral	1	6.2
Moderately unsatisfied	2	12.5
Very unsatisfied	2	12.5
**MHS received in the past**		
Psychological	12	38.8
Psychiatric	7	22.5
Social Work	1	3.2
Professional Counseling	2	6.4
Psychological and Psychiatric	8	25.9
Psychiatric & Social Work	1	3.2
**Type of Therapy**		
Individual Therapy	21	67.9
Family Therapy	2	6.4
Group Therapy	4	12.9
Individual & group therapy	1	3.2
Missing	3	9.6
**Satisfaction with MHS received in the past**		
Very satisfied	9	29
Moderate satisfied	3	9.6
Neutral	5	16.4
Moderately unsatisfied	3	9.6
Very unsatisfied	7	22.5
Missing	4	12.9

Table 3 summarizes mental health services preferences. The majority of the sample (n = 78, 65%) reported being receptive to seeking services if needed and interested in individual therapy (n = 38, 31.6%) and followed by family therapy (n = 14, 11.6%). Most participants reported preferring mental health service appointments during the morning hours (n = 43, 35.8%) followed by any time (n = 30, 25%); the same day of the scheduled oncology appointment (n = 35, 29.2%) followed by any day (n = 33, 27.5%). As for the place where they prefer receiving mental health services, the top 3 choices were the oncology clinic (n = 40, 33.3%), a mental health clinic (n = 17, 14.1%) and at home (n = 6, 5%). Participants were also asked which type of mental health service they would recommend to other cancer patients and the top 3 recommended services were psychological (n = 34, 28.3%), followed by professional counseling (n = 18, 15%) and all services (n = 17, 14.1%).

**Table 3:** Mental health service (MHS) preference (n = 120).

**Variable**	**Frequency**	**%**
**Interested in receiving MHS**		
Yes	78	65
No	17	14.1
Not applicable	12	10
Missing	13	10.8
**Type of therapy**		
Individual therapy	38	31.6
Family therapy	14	11.6
Group therapy	7	5.8
Complementary therapy	3	2.5
Individual and family therapy	3	2.5
Individual and group	1	.8
All	8	6.6
No applicable	28	23.3
Couple	1	.8
Family/ Group	1	.8
Individual/ couple	2	1.6
Individual/ complementary	2	1.6
Missing	12	10
**Preferred times to MHS**		
In the morning	43	35.8
Afternoon	14	11.7
At night	7	5.8
**Variable**	**Frequency**	**%**
Any time	30	25
No applicable	13	10.8
Morning and afternoon	1	.8
Missing	12	10
**Day preferred to MHS**		
Week days	22	18.3
Weekends	6	5
Same day of treatment	35	29.2
Any day	33	27.5
Weekday and day of treatment	2	1.7
No applicable	13	10.8
Missing in system	9	7.5
**Preferred location of MHS**		
Hospital	6	5
Mental health clinic	17	14.1
Oncology office	40	33.3
Phone	4	3.3
Home	6	5
All	6	5
MH clinic/ oncology	3	2.5
Oncology office/ home	3	2.5
No applicable	11	9.16
Hospital/ MH clinic/ Oncology	3	2.5
Phone/ other	1	.8
Oncology/ internet/ home	2	1.6
Oncology/ phone	3	2.5
Missing	12	10
**MHS willing to recommend**		
Psychological	34	28.3
Psychiatric	2	1.7
Social Work	8	6.7
Professional counseling	18	15
Psychological and counseling	7	5.8
Psychological and Psychiatric	7	5.8
Psychological and social	4	3.3
All services	17	14.1
Social Work, counseling/ other	1	.8
Psychologist/ Social work/ counseling	4	3.3
Family counseling	1	.8
Others	4	3.3
Psychiatric/ Counseling	1	.8
Missing	12	10

### Socio-demographic factors associated with mental health service preferences and use

Chi-squares tests were performed to explore associations among socio-demographic variables and mental health service preferences. Next we report associations that were statistically significant. There were significant relationships between preference for using cell phone to receiving MH information and gender, X^2^ (2, n = 111) = 6.72, *p*< .05; as well as preference for using cell phone to receiving MH information and romantic relationship status, X^2^ (2, n = 111) = 7.61, *p*< .05. As such, we found that women (61%) and those living with a romantic partner (67.8%) were more likely to prefer receiving mental health information through the cell phone. We also found significant relations between education and the day preferring mental health services (MHS), X^2^ (15, n = 111) = 30.02, *p*< .05. Participants with no high school degree (36.7%) and those with a college degree (38.5%) preferred the MHS appointment any day, participants with a high school degree (45.5%) preferred having the MHS appointments the same day they have the oncology services, participants with a technical degree preferred any day during the week (40.9%) and participants with a preferred any day of the week. We also found a significant relationship between employment status and preference for type of therapy, X^2^ (15, n = 108) = 40.349, *p*< .000; also, employment status and preferring days of the week X^2^ (4, n = 109) = 9.93, *p*< .05, and employment status and time of the day preferring MH, X^2^ (3, n = 95) = 25.18, *p*< .000. Participants not employed (35.2%) were more likely to prefer individual psychotherapy, have the MHS appointments the same day receiving oncology services (34%) or any day (31%) and during the morning (51.2%). On the other hand, participants reporting being employed at the time of the study preferred receiving MHS during the evening (38.5%) or any time (30.8%) and on weekdays (33.3%).

Chi-squares tests were also performed to explore associations among socio-demographic variables and mental health service use currently, in the past and during lifetime. Next we report associations that were statistically significant. The relationship between past use of MHS and living situation was significant, X^2^ (1, n = 120) = 3.85, *p*< .05, as well as lifetime MHS use and living situation, X^2^ (1, n = 120) = 4.58, *p*< .05. Out of those participant reporting past MHS use (n = 31) and lifetime MHS use (n = 39), those living alone were more like to have used MHS in the past and during their lifetime (71%-past, 72%-lifetime). Additionally, there were significant associations between lifetime MHC use and gender X^2^ (1, n = 120) = 3.63, *p*< .05. Among participant reporting lifetime MHS use (n = 39), women (67%) were more likely than men to have used such services in their lifetime.

Unadjusted and adjusted logistic regressions were performed to assess the relationship and predictive value among sociodemographic factors and lifetime, past and current MHS use (Table 4). As shown in Table 4, unadjusted logistic regression analyses did reveal associations regarding living situation (partnered) and MHSU in their lifetime. Living with someone was a protective factor for not using MHS in their lifetime (OR = 0.36; CI = 0.137–0.938). This relationship remained significant in adjusted regression analyses (OR = 0.28; C1 = 0.08–0.95). On the other hand, other than living situation, the (unadjusted or adjusted) regression models performed did not identified other sociodemographic variables predicting lifetime, past or current mental health service use.

**Table 4:** Unadjusted and adjusted logistic regression models predicting likelihood of using mental health services.

Variables	Unadjusted		Adjusted	
**No lifetime use of MHS (n = 47)**	**OR**	**CI**	**OR**	**CI**
Sex	2.15	0.972–4.77	0.53	0.22–1.28
Living situation (accompanied)	0.36*	0.137–0.938	0.28*	0.08–0.95
Age <60 y/o	0.69	0.318–1.48	0.64	0.26–1.59
Partnered	1.32		0.71	0.25–2.01
Education				
No H.S	0.90	0.308–2.66	1.09	0.27–4.37
H.S.	1.47	0.494–4.39	1.52	0.37–6.21
Tech. degree	0.63	0.19–2.1	0.82	0.06–10.85
Medical Insurance				
Government Ins.	0.20	0.024–1.66	0.13	0.02–1.75
Private Ins.	0.25	0.027–2.41	0.16	0.40–3.83
Income **<** 19K per year	0.99	0.424–2.31	1.24	0.40–3.83
Income not enough	1.19	0.54–2.62	1.3	0.52–3.24
Employed			1.90	0.49–7.45
**No past use of MHS (n = 31)**	**OR**	**CI**	**OR**	**CI**
Sex	2.14	.909–5.076	0.94	0.15–6.06
Living situation (accompanied)	0.38	0.14–1.02	0.27	0.02–4.55
Age <60 y/o	0.80	0.35–1.82	3.59	0.37–20.77
Partnered	1.56	.68–3.54	0.59	0.06–5.58
Education				
No H.S.	0.85	0.27–2.70	5.55	0.14–228.8
H.S.	1.43	0.44–4.70	0.49	0.03–8.00
Tech. degree	0.63	0.19–2.10	0.82	0.06–10.85
Medical Insurance				
Government Ins.	0.30	0.35–2.43	0.52	0.01–24.38
Private Ins.	0.32	0.33–3.04	0.18	0.003–11.5
Income **<** 19K per year	0.84	0.33–2.13	1.37	0.15–12.29
Income not enough			0.65	0.09–4.82
Employed	1.60	0.42–6.03	0.88	0.04–20.77
**No current use of MHS (n = 16)**	**OR**	**CI**	**OR**	**CI**
Sex	2.03	.661–6.27	1.29	0.22–7.5
Living situation (accompanied)	0.4	0.12–1.31	0.18	0.02–1.88
Age <60 y/o	0.86	0.30–2.46	0.16	0.02–1.25
Partnered	0.36	0.12–1.11	0.22	0.02–2.21
Education				
No H.S	1.61	0.31–7.91	0.76	0.02–23.33
H.S.	1.89	0.39–9.22	0.28	0.02–4.44
Tech. degree	0.47	0.12–1.93	0.17	0.01–2.19
Medical Insurance				
Government Ins.	1.2	0.48–2.78	0.07	.002–2.80
Private Ins.			0.17	.003–8.89
Income **<** 19K per year	0.67	0.39–1.15	10.58	0.88–126.66
Income not enough	0.94	0.34–2.71	0.75	0.14–3.96
Employed	0.62	0.16–2.47	0.73	0.08–6.58

*Odds ratios (OR) and 95% confidence intervals (CI) of demographic and mental health factors predicting lifetime*, *past and current use of mental health services*

^#^High School

* p < .05

** p < .01

*** p < .001

## Discussion

The present study aimed to assess preferences, use of and satisfaction with mental health services in a sample of patients with cancer undergoing oncology treatment. It is important to note that the majority of the sample was older than 61 years of age (53.8%, n = 62). This finding is expected considering that a majority of people diagnosed with cancer are older adults.[39] Studies have shown that the population of elderly patients with cancer have particular psychosocial needs that warrant prompt care.[40] For example, a study conducted by Akechi et al.[41] found significant differences between the elderly and younger patients regarding the quality of life in the areas of physical, emotional and social functioning. On the other hand, identified unmet concerns in the elderly patient group were "fear of cancer" (metastasis), "concern for the emotional state of loved ones" and "concern that treatment outcomes are beyond their control".[41] Also, we found that 44.4% of the study sample reported an income equal to or less than $12,000. This finding is not surprising considering Puerto Rico’s economic crisis and the mass migration of the middle class Puerto Ricans to the United States.[42] In fact, this finding coincides with a report published by the Pew Research Center highlighting that 45% of Puerto Rican living in the island are living in poverty levels.[43] Poverty among Puerto Ricans is expected to increase due to Hurricane Maria as income is expected to lower by 21% in the next fifteen years.[44]

We found that 11.1% (n = 13) of the sample reported that at some point they felt the need to receive mental health services and did not use it. This finding is worthy of analysis because it represents a group of people who have internalized the need for professional mental health care for some reason, he/she reframed from doing so. This coincides with a systematic review of literature published by Cabaza, Zayas and Hansen[45], which found that, compared to Non-Latino Whites, the Latino population tends to use mental health services less frequently, seek more frequently the services of primary physicians to attend to their mental health problem and, therefore, do not receive mental health treatment as recommended by clinical guidelines.(45) The study also reveals that Latinos living in the United States and Puerto Rico who suffer from mental health problems tend to use the combination of primary care services and the support of a couple who advise them to deal with their psychiatric condition.[45]

Overall, most participants (66.7%, n = 78) reported interest in receiving mental health services if necessary. This is important because it suggests an apparent openness and acceptance towards mental health services. However, these data must be interpreted with care because it depends on the perception of need for mental health services. Although self-assessment of mental health status may be a predictor of the use of mental health services in Hispanics, there is a high proportion of people with symptoms of a mental health disorder who do not seek services because they feel that they do not help.[46] A systematic review conducted by Serephia-Derr[47] concluded that potential cultural barriers to mental health service use among the immigrant population in the United States are preferences for informal mental health support (friends, religious leader or family member) and stigma.

Based on regression analyses, we found that living with someone was a protective factor for not using mental health services throughout participants’ lifetime. This finding may be partially explained by two factors including the provision of social support or the *Familism* cultural value known to be common among Hispanics. For instance, a meta-analysis conducted by Harandi and collaborators found moderate significant correlations between social support and mental health.[48] On the other hand, a study about mental health treatment utilization among United States’ military veterans found that participants (n = 100) who received a combination of psychotherapy and pharmacotherapy for a mental health illness reported less post-deployment social support.[49] Moreover, a multi-ethnic study investigating the role of social support in the relationship between stress and mental health found that, compared to other ethnic groups (non-Hispanic Whites, African Americans, Asian Americans), social support acted as a buffering effect only among Hispanic Mexican Americans.[49] Even though social support may be a protective factor for a mental health illness, the cultural value Familism may prevent Hispanic patients from seeking mental health services if needed.[50] Instead mental health services, Hispanic tend to prefer informal (e.g. self-help groups, online groups) or religious services (e.g. religious leader, healer).[50] However, Hispanics may tend to seek mental health services when close family/friends support networks advise the use of professional mental health services.[50]

Findings suggest that it is important to incorporate service satisfaction assessment measures. Of note, mental health service satisfaction has been positively associated with therapeutic alliance.[51] We urge that assessment of satisfaction with services be anonymous to reduce potential bias associated with social desirability. Also, the quantitative aspect of the satisfaction survey could be supplemented with open ended prompts for patients to give their suggestions for how the service could be improved to better match patient needs.

The current study had some potential limitations. For instance, since the study was meant to be descriptive, the correlation analyses made are intended to be exploratory rather than for hypothesis testing. Also, the small sample size of some sub-groups (e.g., MHS satisfaction among those receiving such service at the time of the study, n = 16; moderately satisfied = 5) may affect the interpretability of such results. Future studies should inquire about satisfaction of MHS among cancer patients should either include a sub-sample of participants consumers of such services or recruit only participants who are or have been MHS users. Other potential limitation is that we included all cancer types and stages, thus, limiting representation of specific cancer sites. Future studies of this kind should either focus on one cancer site or stratify the sample by cancer site and stage. Another limitation is that the non-probabilistic sample size prevents generalization of results. Also, the study included a only Puerto Rican Hispanic patients and some of it results may not apply to other sub-ethnicities of Hispanic cancer patients.

### Conclusion

Participants’ MHS preferences reported in this study supports the integration of mental health services with the clinical oncological practice. Based on our findings, if we sketch a profile of preferred mental health services reported by participants it would be the following: mental health services provided at the oncology clinic (34.2%, n = 40), with appointments scheduled during the morning (35.9%, n = 42) preferably during the same day they receive the oncology services (29.9%, n = 35) or any day (28.2%, n = 33) and the necessary frequency (52.1%, n = 61). Future mixed methods studies are needed to explore other personal and system barriers for mental health service use. For example, a personal barrier may be beliefs and attitudes towards mental health services as well as the perception of access to services. Other system barriers may be economic factors and access to mental health services.

## Supporting information

S1 Dataset (SAV)
